# Supplementary material for: Bespoke magnetic field design for a magnetically shielded cold atom interferometer
Source: Sci Rep. 2022 Jun 22;12:10520. doi: 10.1038/s41598-022-13979-4 (PMC9217970; doi:10.1038/s41598-022-13979-4)
Supplement: Supplementary file 1 — Supplementary Information. [file 41598_2022_13979_MOESM1_ESM.pdf]

Supplementary Information

S.1 Mathematical appendix

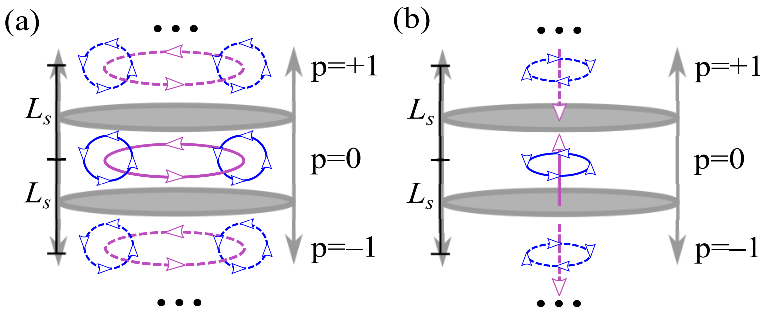

Figure S.1. Mirror image pseudocurrents[55] (purple dashed) generated by symmetric (a) azimuthal and (b) axial current flows (purple solid) inside the shield presented in Fig. 1. The index p represents the reflection number for $p \in \mathbb{Z}$. The reflections extend along an infinite cylinder but only $p=-1$ (bottom) and $p=+1$ (top) reflections are illustrated. To satisfy the axial boundary condition, the azimuthal pseudocurrents have the same flow direction as those on the coil, whereas the axial flows are reversed for reflections where p is odd. Simplified flux lines (blue) illustrate how this satisfies (2) at the end-caps.

Equation (1) is solved in Ref. 40 by separating the boundary conditions in equation (2) at the radial shield wall and end caps. The boundary condition at the radial shield wall is satisfied by matching the magnetic field generated by the coil and pseudocurrent over an infinitely long cylinder at $\rho=\rho_s$. The boundary condition at the end caps is satisfied by applying an infinite sum of mirror images[55]. The magnetic field parallel to the end cap surfaces is nulled by the infinite summation of reflections of the currents on the coil. A simplified illustration of this is provided in Fig. S.1. The total magnetic field is

$$B_\rho\left(\rho,\phi,z\right)=\frac{i\mu_0\rho_c}{2\pi}\sum_{m=-\infty}^{\infty}\sum_{p=-\infty}^{\infty}\int_{-\infty}^{\infty}\mathrm{d}k\; k e^{im\phi}e^{ikz}I'_m(|k|\rho)R_m(k)J_\phi^{mp}(k)\;, \tag{S.1}$$

$$B_\phi\left(\rho,\phi,z\right)=-\frac{\mu_0\rho_c}{2\pi\rho}\sum_{m=-\infty}^{\infty}\sum_{p=-\infty}^{\infty}\int_{-\infty}^{\infty}\mathrm{d}k\; m\frac{|k|}{k}e^{im\phi}e^{ikz}I_m(|k|\rho)R_m(k)J_\phi^{mp}(k)\;, \tag{S.2}$$

$$B_z\left(\rho,\phi,z\right)=-\frac{\mu_0\rho_c}{2\pi}\sum_{m=-\infty}^{\infty}\sum_{p=-\infty}^{\infty}\int_{-\infty}^{\infty}\mathrm{d}k\; |k|e^{im\phi}e^{ikz}I_m(|k|\rho)R_m(k)J_\phi^{mp}(k)\;, \tag{S.3}$$

where

$$J_\phi^{mp}(k)=\frac{1}{2\pi}\int_0^{2\pi}\mathrm{d}\phi'\; e^{-im\phi'}\int_{-\infty}^{\infty}\mathrm{d}z'\; e^{-ikz'}\; J_\phi\left(\phi',(-1)^p\left(z'-pL_s\right)\right)\;, \tag{S.4}$$

is the Fourier transform of the pth reflected image current formed by the method of mirror images and

$$R_m(k)=K'_m(|k|\rho_c)-\frac{I'_m(|k|\rho_c)K_m(|k|\rho_s)}{I_m(|k|\rho_s)}\;, \tag{S.5}$$

where $I_\nu(z)$, $I'_\nu(z)$, and $K_\nu(z)$ are the modified Bessel function of the first kind, its derivative with respect to z, and the modified Bessel function of the second kind, of order ν, respectively. At this point, it should be noted that substituting a constant axial current density, $J_\phi=I/L_c$, into equation (S.4) and setting that the coil and shield are of the same length, $L_c=L_s$, generates the result that the B_z field is entirely uniform[40]. This is equivalent to a perfect solenoid inside a closed perfect magnetic conducting shield of equal length to the solenoid.

Because the coil is placed symmetrically inside the shield, we can pose a Fourier series formulation of $J_\phi\left(\phi',z'\right)$ that satisfies the method of mirror images (Fig. S.1) and represents a complete basis of modes of current flow on the coil,

$$J_\phi\left(\phi',z'\right)=\left(H\left(z'-\frac{L_c}{2}\right)-H\left(z'+\frac{L_c}{2}\right)\right)\left[\sum_{n=1}^{N}W_{n0}\sin\left(n\pi\left(\frac{z'}{L_c}-\frac{1}{2}\right)\right)\right.$$
$$\left.+\sum_{n=1}^{N}\sum_{m=1}^{M}\left(W_{nm}\cos(m\phi')+Q_{nm}\sin(m\phi')\right)\cos\left(n\pi\left(\frac{z'}{L_c}-\frac{1}{2}\right)\right)\right]\;, \tag{S.6}$$

where $H(x)$ is the Heaviside step function and $(W_{n0},\ W_{nm},\ Q_{nm})$ are Fourier coefficients. By substituting equation (S.6) into equations (S.1–S.3) and integrating over the surface of the coil, we relate the Fourier coefficients to the magnetic field at any point inside the shield,

$$B_\rho(\rho,\phi,z) = \sum_{n=1}^{N} W_{n0} F_n(\rho,z) + \sum_{n=1}^{N}\sum_{m=1}^{M}\left(W_{nm} G_{nm}^{w}(\rho,\phi,z) + Q_{nm} G_{nm}^{q}(\rho,\phi,z)\right), \tag{S.7}$$

$$B_\phi(\rho,\phi,z) = \sum_{n=1}^{N}\sum_{m=1}^{M}\left(W_{nm} H_{nm}^{w}(\rho,\phi,z) + Q_{nm} H_{nm}^{q}(\rho,\phi,z)\right), \tag{S.8}$$

$$B_z(\rho,\phi,z) = \sum_{n=1}^{N} W_{n0} D_n(\rho,z) + \sum_{n=1}^{N}\sum_{m=1}^{M}\left(W_{nm} S_{nm}^{w}(\rho,\phi,z) + Q_{nm} S_{nm}^{q}(\rho,\phi,z)\right), \tag{S.9}$$

with

$$F_n(\rho,z) = -2\mu_0\rho_c n L_c \sum_{p=-\infty}^{\infty} I_0'\left(\left|\frac{\pi p}{L_s}\right|\rho\right)\frac{R_{0p}\ p}{n^2L_s^2 - L_c^2p^2}\begin{pmatrix}\sin\left(\frac{\pi p z}{L_s}\right)\cos\left(\frac{\pi p L_c}{2L_s}\right)(1+(-1)^p)\\ \cos\left(\frac{\pi p z}{L_s}\right)\sin\left(\frac{\pi p L_c}{2L_s}\right)(1-(-1)^p)\end{pmatrix}, \tag{S.10}$$

$$G_{nm}(\rho,z) = \frac{2\mu_0\rho_c L_c^2}{L_s}\sum_{p=-\infty}^{\infty} I_m'\left(\left|\frac{\pi p}{L_s}\right|\rho\right)\frac{R_{mp}\ p^2}{n^2L_s^2 - L_c^2p^2}\begin{pmatrix}\cos\left(\frac{\pi p z}{L_s}\right)\cos\left(\frac{\pi p L_c}{2L_s}\right)((-1)^p-1)\\ \sin\left(\frac{\pi p z}{L_s}\right)\sin\left(\frac{\pi p L_c}{2L_s}\right)((-1)^p+1)\end{pmatrix}, \tag{S.11}$$

$$G_{nm}^{w}(\rho,\phi,z) = \cos(m\phi)\,G_{nm}(\rho,z)\ ,\quad G_{nm}^{q}(\rho,\phi,z) = \sin(m\phi)\,G_{nm}(\rho,z)\ , \tag{S.12}$$

$$H_{nm}(\rho,z) = -\frac{2\mu_0\rho_c L_c^2}{\pi\rho}\sum_{p=-\infty}^{\infty} I_m\left(\left|\frac{\pi p}{L_s}\right|\rho\right)\frac{R_{mp}\ |p|}{n^2L_s^2 - L_c^2p^2}\begin{pmatrix}\cos\left(\frac{\pi p z}{L_s}\right)\cos\left(\frac{\pi p L_c}{2L_s}\right)((-1)^p-1)\\ \sin\left(\frac{\pi p z}{L_s}\right)\sin\left(\frac{\pi p L_c}{2L_s}\right)((-1)^p+1)\end{pmatrix}, \tag{S.13}$$

$$H_{nm}^{w}(\rho,\phi,z) = m\sin(m\phi)\,H_{nm}(\rho,z)\ ,\quad H_{nm}^{q}(\rho,\phi,z) = -m\cos(m\phi)\,H_{nm}(\rho,z)\ , \tag{S.14}$$

$$D_n(\rho,z) = -2\mu_0\rho_c n L_c \sum_{p=-\infty}^{\infty} I_0\left(\left|\frac{\pi p}{L_s}\right|\rho\right)\frac{R_{0p}\ |p|}{n^2L_s^2 - L_c^2p^2}\begin{pmatrix}\cos\left(\frac{\pi p z}{L_s}\right)\cos\left(\frac{\pi p L_c}{2L_s}\right)((-1)^p+1)\\ \sin\left(\frac{\pi p z}{L_s}\right)\sin\left(\frac{\pi p L_c}{2L_s}\right)((-1)^p-1)\end{pmatrix}, \tag{S.15}$$

$$S_{nm}^{w}(\rho,\phi,z) = \cos(m\phi)\,S_{nm}(\rho,z)\ ,\quad S_{nm}^{q}(\rho,\phi,z) = \sin(m\phi)\,S_{nm}(\rho,z)\ , \tag{S.16}$$

$$S_{nm}(\rho,z) = \frac{2\mu_0\rho_c L_c^2}{L_s}\sum_{p=-\infty}^{\infty} I_m\left(\left|\frac{\pi p}{L_s}\right|\rho\right)\frac{R_{mp}\ |p|p}{n^2L_s^2 - L_c^2p^2}\begin{pmatrix}\sin\left(\frac{\pi p z}{L_s}\right)\cos\left(\frac{\pi p L_c}{2L_s}\right)(1-(-1)^p)\\ \cos\left(\frac{\pi p z}{L_s}\right)\sin\left(\frac{\pi p L_c}{2L_s}\right)(1+(-1)^p)\end{pmatrix}, \tag{S.17}$$

where

$$R_{mp} = K_m'\left(\left|\frac{\pi p}{L_s}\right|\rho_c\right) - \frac{I_m'\left(\left|\frac{\pi p}{L_s}\right|\rho_c\right)K_m\left(\left|\frac{\pi p}{L_s}\right|\rho_s\right)}{I_m\left(\left|\frac{\pi p}{L_s}\right|\rho_s\right)}, \tag{S.18}$$

and the vector $\begin{pmatrix}a\\b\end{pmatrix}$ denotes the function for either odd, a, or even, b, n.

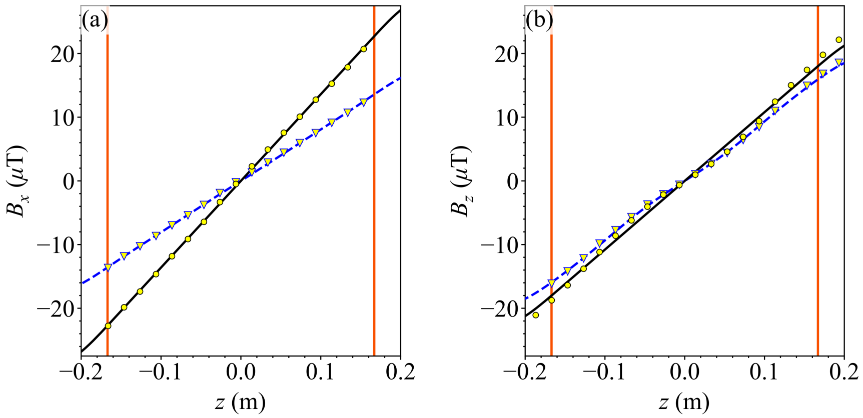

Figure S.2. (a) Transverse magnetic field generated by the constant transverse gradient $\mathrm{d}B_x/\mathrm{d}z$ coil (current $I = 500$ mA) with/without the magnetic shield (black/dark blue) evaluated along the z-axis of the standalone shield. Scatter points (yellow circle/triangle with black/dark blue outline) show measured data and lines show theoretical field profiles calculated analytically from the theoretical model (solid black) or numerically via the Biot–Savart law (dashed dark blue). Overlaid lines (solid red) show the edges of the optimisation region. (b) Axial magnetic field generated by the constant axial gradient $\mathrm{d}B_z/\mathrm{d}z$ coil (current $I = 500$ mA) with/without the magnetic shield (black/dark blue) evaluated along the z-axis of the interferometer. Labelled identically to (a).

S.2 Characterisation of the constant linear gradient field coils

In Fig. S.2, the transverse and axial fields generated by the constant transverse and axial linear gradient coils, $\mathrm{d}B_x/\mathrm{d}z$ and $\mathrm{d}B_z/\mathrm{d}z$, respectively, are presented along the z-axis. These measurements were taken using a duplicate mounting structure of the sensor without the atomic package or other experimental systems. The experimentally measured fidelities of these coils, $(\mathrm{d}B_x/\mathrm{d}z)^{\mathrm{RMS}} = 2\%$ and $(\mathrm{d}B_z/\mathrm{d}z)^{\mathrm{RMS}} = 6\%$, respectively, (Table 1 in the main text) are in line with many free-space optimisation techniques, but are diminished compared to the other coils in the former. This is because fewer contour levels were used to represent the gradient coils, so that the former could be manufactured easily. The RMS errors for the gradient coils are quoted to a lower precision than for the uniform field generating coils due to increased sensitivity to the axial position of the magnetometer in the calculation of the gradients.

S.3 Comparing the uniform transverse field to that generated by other coil systems

In Fig. S.3a, we present the rectangular coils designed previously to generate a uniform transverse field in the atom interferometer. These coils were housed directly on the vacuum system, limiting their length. In Fig. S.3c, the uniformity of the transverse field generated by these coils, along the z-axis of the atom interferometer, is calculated from the numerical simulations and is compared to equivalent simulations of the uniform B_x field coil housed on the 3D-printed coil (Fig. 5). Along the z-axis of the optimisation region, the field generated by the uniform B_x coil deviates by 0.148%, whereas the field generated by the rectangular coils deviates by 41.4%.

Now, let us also compare the performance of the optimised coil to a comparable coil designed using standard techniques. In Ref. 31, Hosoya and Goto solve for the scalar potential on the interior surface of an infinitely long high permeability magnetic shield to generate uniform axial and transverse fields. They then design coil systems to mimic these scalar potentials. Their solution to generate a uniform transverse field, using a dense network of wires, is referred to as a '$2n$ coil'[31]. In this design, axial wires are positioned around a cylinder to emulate the surface current distribution where the axial current density is proportional to the cosine of the azimuthal coordinate. Applying the model presented in the main text, this is equivalent to the contours of the streamfunction proportional to the sine of the azimuthal angle. This coil is represented schematically in Fig. S.3b. Here, we have constructed the '$2n$ coil' using saddles, as other arrangements where the axial wires are connected directly across the cylinder would interfere with the atom interferometer. The coil is generated with an equal number of contour levels to the uniform B_x coil, i.e., $n = 15$, and is housed on a cylinder of the same dimension as the 3D-printed coil former. To make the design realistic, as the azimuthal extent of each saddle reduces, we shorten its axial extent sequentially by 0.90 mm, following the wire diameter used in the manufacture of the uniform B_x coil.

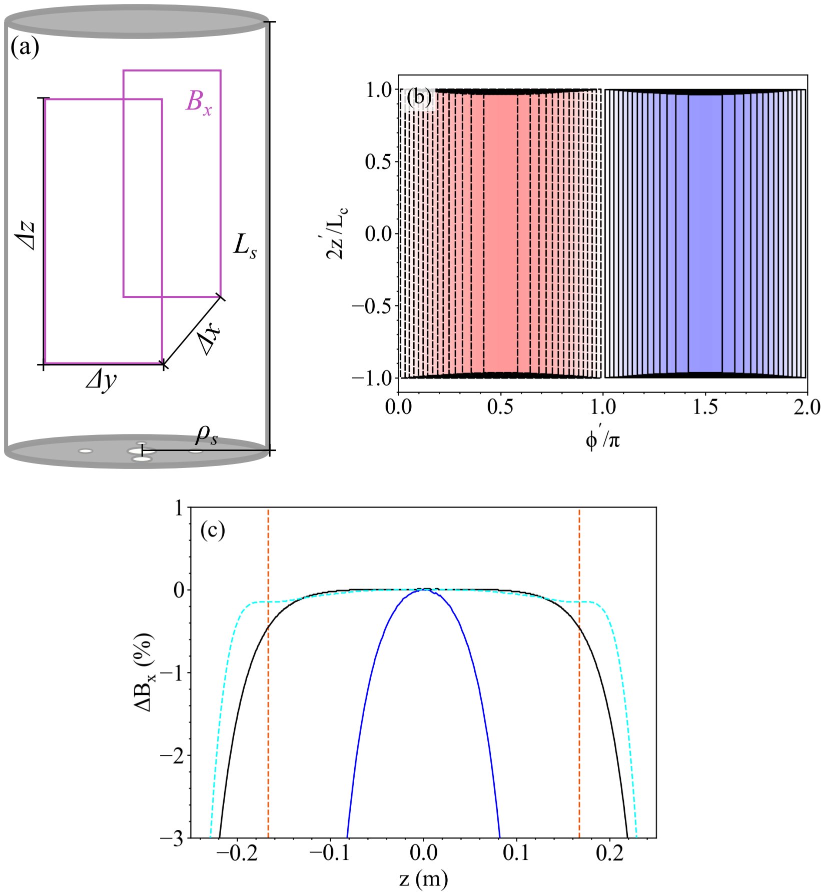

Figure S.3. (a) Schematic diagram of the previously used uniform B_x field-generating compensation coil (purple) inside the cylindrical magnetic shield (grey) of the interferometer of radius ρ_s and length L_s. The previously used coil consists of a rectangular coil pair of width $\Delta y = 100$ mm and height $\Delta z = 350$ mm, separated by $\Delta x = 171$ mm. (b) Normalised streamfunction, calculated using equation (4), at $N_{\text{contours}} = 30$ levels, with $W_{11} = 1$ and all other Fourier coefficients set to zero, on the surface of a coil of length L_c (blue and red shaded regions correspond to the flow of current in opposite directions and the intensity shows the streamfunction magnitude). The discretised pattern is analogous to a '$2n$ coil' from Ref. 31 with $n = 15$. (c) Deviation from perfect uniformity of the normalised transverse field, $\Delta B_x = B_x - 1$, along the z-axis, generated by the optimised B_x coil in Fig. 4a (cyan dashed), coil in (a) (blue solid), and coil in (b) (black solid), in-situ within the atom interferometer (Fig. 1). Overlaid lines (solid red) show the edges of the optimisation region. This data is simulated numerically using COMSOL Multiphysics Version 5.3a.

In Fig. S.3c, the uniformity of the transverse magnetic field generated by the '$2n$ coil' is simulated numerically. The fidelity of the uniform field is vastly improved compared to the rectangular coil but is diminished compared to the uniform B_x coil, particularly at the edge of the optimisation region. The transverse field deviates by a maximum of 0.457% in the optimisation region. This is because the coil does not account for the finite length of the coil cylinder or magnetic shield, or the separation between them, in its design. As well as this, the total wire length is 54.0 m, which is 21% longer than the uniform B_x coil.
